# Tuberculosis Notification in Jordan, 2016–2020

**DOI:** 10.3390/epidemiologia4030028

**Published:** 2023-07-04

**Authors:** Yousef Khader, Hiba Abaza, Srinath Satyanarayana, Ahmad Saleh Abu Rumman, Mohamad Nihad Alyousfi

**Affiliations:** 1 Center of Excellence for Applied Epidemiology, Global Health Development, Eastern Mediterranean Public Health Network, Amman 11195, Jordan; nehad.alyousfi@gmail.com; 2 Migration Health Division, International Organization for Migration (IOM), Amman 11953, Jordan; habaza@iom.int (H.A.); drsrinaths@gmail.com (S.S.); 3 Department of Chest Disease and Foreigners, Jordan Ministry of Health, Amman 11118, Jordan; dr_rumman@yahoo.com

**Keywords:** tuberculosis, notification, trend, epidemiology

## Abstract

The burden of tuberculosis (TB) in Jordan is largely unknown due to the paucity of high-quality data, under-reporting, and a lack of good quality vital registration system. This study aimed to assess the characteristics of TB patients in Jordan, determine the TB notification rate and assess the trend of TB notification in Jordan between 2016 and 2020. **Methods:** This study analyzed the TB Surveillance data in Jordan for the period 2016–2020. The obtained data included information on age, gender, nationality, marital status, date of symptoms onset and date of diagnosis, and site of TB. **Results:** During the period 2016–2020, a total of 1711 patients (989 women and 722 men) were diagnosed with and treated for tuberculosis. The mean (SD) age of patients was 30.1 (17.2) years. Almost half of them (48.4%) were Jordanians. The majority of non-Jordanian patients were from Syria, Philippines, and Bangladesh. Two thirds of patients (66.0%) had pulmonary TB and 34.0% had extra-pulmonary TB. Almost half (50.7%) of the patients were diagnosed within one month of the symptoms’ onset. The average annual TB notification rate during 2016–2020 was 3.32 per 100,000 pop (4.08 per 100,000 women and 2.64 per 100,000 men). The average annual standardized notification rate was 4.13 per 100,000 pop (4.52 per 100,000 women and 3.52 per 100,000 men). The overall age-standardized notification rate increased from 3.88 per 100,000 pop in 2016 to 4.58 per 100,000 pop in 2019 and declined to 2.46 per 100,000 pop in 2020. The trend in TB notification differed significantly according to gender. While the notification increased in the last three years among women, it decreased significantly among men. **Conclusions:** While TB notification increased in the last three years among women, it decreased significantly among men. There is a need to ensure that the national TB plans set clear targets for reducing the burden of TB.

## 1. Introduction

Tuberculosis (TB) is one of the top 10 causes of death worldwide and the leading cause of death from a single infectious agent [1]. TB is a severe chronic bacterial infection caused by Mycobacterium tuberculosis which spreads by coughing or sneezing. The disease typically affects the lungs (Pulmonary TB) but can also affect other sites (extrapulmonary TB). According to the global tuberculosis report 2022 [2], an estimated 10.6 million people had TB in 2021, an increase of 4.5% from 10.1 million in 2020. The TB incidence rose by 3.6% between 2020 and 2021, reversing declines of about 2% per year for most of the previous 2 decades. The burden of drug-resistant TB (DR-TB) was also estimated to have increased between 2020 and 2021, with 450,000 new cases of rifampicin-resistant TB in 2021. Globally, the estimated number of deaths from TB increased between 2019 and 2021, reversing years of decline between 2005 and 2019. In 2021, there were an estimated 1.6 million deaths. This was up from best estimates of 1.5 million in 2020 and 1.4 million in 2019, and back to the level of 2017. The number of TB cases is still unacceptably high knowing that most cases would be curable if better diagnostic tools and correct treatment are available [3,4]. About 85% of people who develop TB disease can be successfully treated with a 6-month drug regimen [1]. Completing tuberculosis treatment is essential for personal health, preventing transmission, minimizing the risk of drug resistance, and contributing to public health control efforts.

Sustainable Development Goal 3 identifies ending the TB epidemic by 2030 under target 3 [5]. The Eastern Mediterranean Region (EMR) has an estimated TB incidence of 116 per 100,000. The main challenges facing TB care in the EMR include limited infrastructure, human capacity, funds, use of new diagnostics tools and new medicines, and involvement of all stakeholders and communities in TB care and control [6].

One of the interventions for TB infection prevention and control is the vaccination of children with the Bacille Calmette-Guérin (BCG) vaccine, which can confer protection, especially from severe forms of TB in children. Global BCG coverage was 88% in 2019, close to the highest coverage of any vaccination [7]. The Jordan Population and Family and Health Survey 2017–2018 [8] showed that 93% of children (12–23 months old) have received the BCG ((Jordanian (93.7%), Syrian (90.0%), and other nationalities (80.5%)). Although the implementation of the BCG vaccine against TB is widespread in high-TB-burden countries, BCG vaccination policies in low-burden countries vary [9,10].

The incidence of TB in Jordan fluctuated substantially in recent years and it decreased between 2000—2019 period ending at 6 cases per 100,000 people in 2019 [11]. The health system in Jordan is a complex combination of three major sectors: public, private, and charity. The public sector consists of two major public programs that finance and provide care: the Ministry of Health (MoH) and Royal Medical Services (RMS). Primary care is delivered through a network of facilities under MoH including 109 comprehensive health centers (CHCs), 374 primary health centers (PHCs), and 186 branch or village health centers (VHCs). Primary, secondary and some tertiary health care services are available to all registered refugees from all nationalities at the non-insured Jordanian rate at public health centers and Governmental hospitals. The National TB Program (NTP) is a national entity with the Chest Disease and Migrant Health Directorate in Amman City at the central level and 12 peripheral chest centers throughout the country. The Chest Disease Division, under the Assistant Secretary General of Primary Health Care, is responsible for planning, coordinating, supplying, and supervising the country’s TB control activities.

The NTP reached the Millennium Development Goal (MDG) for TB reduction in 2011 and was preparing to shift to TB elimination. However, TB elimination planning has been disrupted due to the influx of Syrian refugees. The continuing conflict in Syria resulted in devastating health consequences [12,13], particularly for those with diseases that need long-term treatment like TB. Crisis-affected populations have elevated TB incidence rates and delayed TB treatment, compared with general populations [14]. The International Organization for Migration (IOM) has supported the NTP program in Jordan since 2012 to enhance the detection and treatment of TB among Syrian refugees. Syrian refugee cases constituted 24.4% of all TB cases in 2013 when Syrian refugees made up 6.8% of Jordan’s population, and 13.8% of TB cases in 2015 when Syrians made up 8.3% of the total population.

The burden of TB in Jordan is largely unknown due to the paucity of high-quality data, under-reporting, and a lack of good quality vital registration system [3]. This study aimed to assess the characteristics of TB patients in Jordan, determine the TB notification rate and assess the trend of TB notification in Jordan between 2016 and 2020.

## 2. Methods

This study analyzed the TB Surveillance data in Jordan for the period 2016–2020. This is a secondary data analysis that doesn’t carry potential risks for patients, thus, no informed consent was obtained. Still, this research received the Institutional Review Board (IRB) approval from the Jordan Ministry of Health and written permission from the National Tuberculosis Program (NTP) in Jordan.

The TB Surveillance System in Jordan is passive and national. It collects data on TB patients including Jordanian, Syrian, and migrant patients. The Chest Diseases and Migrants Health Directorate in Amman receives information about any newly diagnosed cases from all health facilities in the country through a monthly report from peripheral TB centers, and then these cases are followed up in the peripheral centers regarding the treatment and the outcome. TB cases are registered and notified according to well-established case definitions. TB case definitions are divided into two groups: a. bacteriologically confirmed TB case: It is a TB case from whom a biological specimen is positive by smear microscopy, culture, or WHO-approved rapid test, such as GeneXpert MTB/RIF assays. b. clinically diagnosed TB case: A case that does not fulfill the criteria for bacteriological confirmation but has been diagnosed with active TB by a medical practitioner who has decided to give the patient a full course of TB treatment. This definition includes cases diagnosed based on X-ray abnormalities or suggestive histopathology evidence and extrapulmonary cases without laboratory confirmation. Clinically diagnosed cases subsequently found to be bacteriologically positive, before or after starting TB treatment, are reclassified as bacteriologically confirmed.

The TB Surveillance System dataset is a case-based format. Paper-based data collection is used. The obtained data included information on age, gender, nationality, marital status, date of symptoms onset and date of diagnosis, and site of TB.

The data analysis was conducted using SPSS 24. Data were presented using mean, standard deviation, and percentages. The characteristics of patients were compared between men and women using the chi-square test. Age-standardized notification of TB was calculated. Expected TB cases for 2020 were estimated by fitting the overdispersed Poisson generalized linear models to the monthly cases for the period of 2016–2019. The model included month and year as variables to capture seasonality and adjust for annual trends. The month was entered in the model as a categorical variable. A *p*-value of less than 0.05 was considered statistically significant.

## 3. Results

### 3.1. Patients’ Characteristics

During the period 2016–2020, a total of 1711 patients (989 women and 722 men) were diagnosed with and treated for tuberculosis. Of those, 14 cases were classified as relapses. The mean (SD) age of patients was 36.1 (17.2) years, being significantly (*p* < 0.001) higher for men compared to women (39.4 (19.3) years vs. (33.7 (15.1) years). Almost three-quarters of women (76.0%) and 54.6% of men were aged between 20 and 49 years. Table 1 shows their demographic and clinical characteristics.

**Figure 1:**
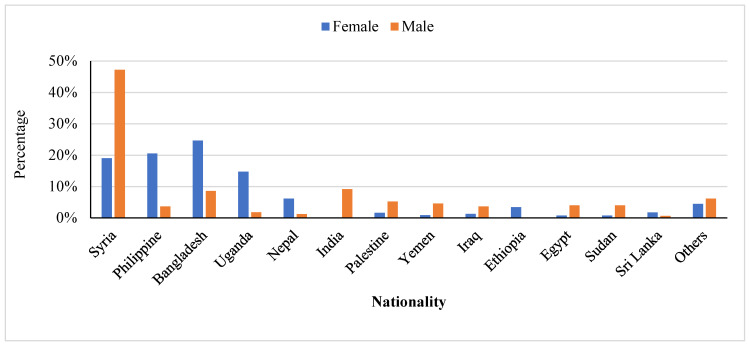
The distribution of tuberculosis cases among non-Jordanians according to gender, 2016–2020.

Almost half of the patients (48.4%) were Jordanians and 51.6% were non-Jordanians. The distribution of non-Jordanians according to nationality is shown in Figure 1. The majority of non-Jordanian patients were from Syria, Philippines, and Bangladesh. The characteristics of patients with TB according to nationality are shown in Table 2. Almost half of the Jordanian patients (52.2%) and 40.8% of Syrian patients were women. On the other hand, TB patients of other nationalities were predominantly women (72.3%).

**Figure 2:**
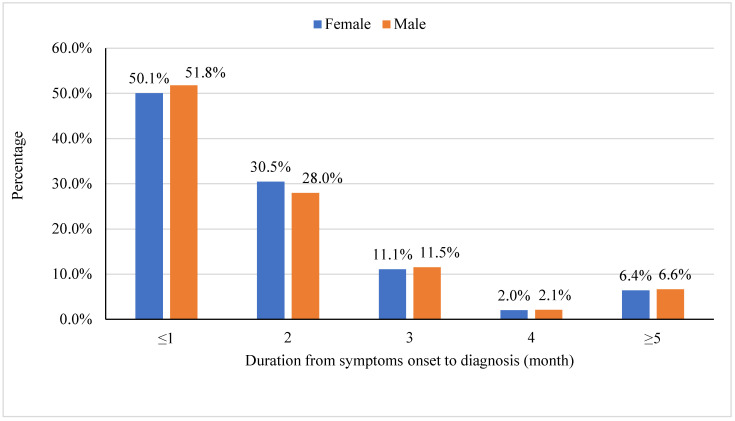
The duration from symptoms onset to diagnosis (month) for patients with TB, 2016–2020.

Two thirds of patients (66.0%) had pulmonary TB and 34.0% had extra-pulmonary TB. While less than half (48.3%) of Jordanian patients had pulmonary TB, the majority of Syrian patients (79.6%) and patients of other nationalities (83.9%) had pulmonary TB. Almost half of the patients were diagnosed within one month of the symptoms’ onset (Figure 2).

### 3.2. TB Notification

The average annual crude TB notification rate during 2016–2020 was 3.32 per 100,000 pop (4.08 per 100,000 women and 2.64 per 100,000 men). Table 3 shows the gender- and age-specific TB notification rates during the period 2016–20120. The highest notification rate among women was in the fourth decade of life and among men aged ≥70 years. The average annual standardized rate was 4.13 per 100,000 pop (4.52 per 100,000 women and 3.52 per 100,000 men).

### 3.3. The Trend of TB Notification

**Figure 3:**
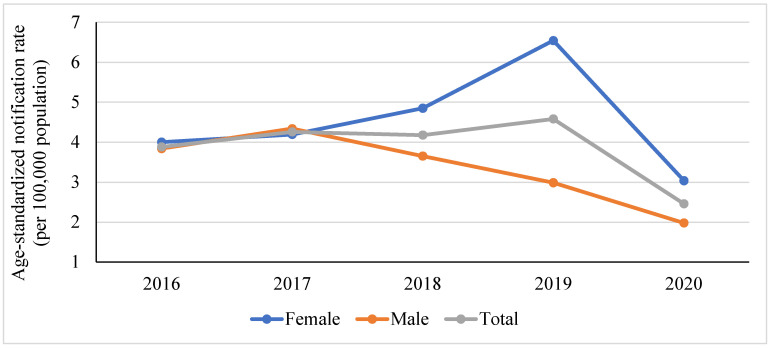
The age-standardized notification rate of tuberculosis in Jordan, 2016–2020.

The overall age-standardized notification rate increased from 3.88 per 100,000 pop in 2016 to 4.58 per 100,000 pop in 2019 and declined to 2.46 per 100,000 pop in 2020 (Figure 3). The trend in TB notification rate differed significantly according to gender. While the notification rate increased in the last three years among women, it decreased significantly among men. The decline in the notification in 2020 reflects the impact of COVID-19 on the diagnosis and management of TB. In 2020, a total of 243 patients (144 women and 99 men) were diagnosed with TB. Using Poisson generalized linear models, the predicted number of cases in 2020 was estimated at 502 cases (289 women and 213 men) which results in a rate of 5.43 per 100,000 of the population.

## 4. Discussion

This study showed that the standardized notification rate of TB increased by 18% between 2016 and 2019. In 2020, it declined by 46% relative to the rate in 2019. The decline in the registered TB cases in 2020 is consistent with what happened globally during the time of the COVID-19 pandemic and restrictive measures. The COVID-19 pandemic continues to have a damaging impact on BCG vaccination, access to TB diagnosis and treatment, and the burden of TB disease. The most obvious and immediate impact was a large global drop in the reported number of people newly diagnosed with TB. From a peak of 7.1 million in 2019, this fell to 5.8 million in 2020 (–18%), back to the level last seen in 2012. In 2021, there was a partial recovery, to 6.4 million [2]. Lockdown-related interruptions can lead to enduring increases in TB burden. However, these negative effects can be mitigated with the fast continuation of TB services, and well-designed interventions that are implemented as soon as restrictions are lifted [15]. Depending on its pre-COVID-19 readiness, a country’s TB program might take long time to restore TB services to normal after the lockdown. As well, there might be a reluctance to seek care among those with TB symptoms due to the fear and stigma caused by getting infected with COVID-19 [16]. The healthcare system exhaustion during the pandemic could explain the negative effect on TB. Countries with a high TB burden like India reported declining TB notifications during 2020 [17,18]. Integrating TB case finding into COVID-19 screening, testing, tracing, and monitoring systems and enhancing access to care would be an appropriate strategy to increase TB notification in the country [19]. Likewise, including knowledge and awareness of TB in the COVID-19 community and health care providers’ education about infection prevention, protective health behaviors, and stigmatization could contribute to reducing TB transmission.

Two thirds of TB cases in Jordan were pulmonary TB and one-third of cases were extrapulmonary TB. This finding is consistent with previous TB statistics in Jordan [15]. The proportion of pulmonary TB is considered high when it is compared with the WHO global data, which indicates that extrapulmonary TB without associated lung involvement accounts for 15–20% of TB in populations with a low prevalence of HIV infection like Jordan. While in populations with a high prevalence of HIV infection, the proportion of cases with extrapulmonary TB is higher [20].

Women had a more than 50% higher notification of TB compared to men. The highest notification rate was seen among women in their thirties and men aged 70 years or above. This finding is similar to the findings of other studies [21,22,23] that highlight the role of socioeconomic and cultural factors in determining gender disparities in TB risk, progression, and treatment. Gender differentials in some contexts may lead to differential exposure to tuberculosis bacilli. Furthermore, the general health/nutritional status of TB-infected persons affects the disease progression and recovery. Gender differences also exist in women’s access to and compliance with treatment. In low-income countries, TB kills more women than all causes of maternal mortality combined [24]. Young women have up to 34% higher risk of progression to TB disease than men due to reduced immunity associated with the stresses of pregnancy [25]. In women, a new infection with TB carries a greater risk of progressing to TB disease more rapidly. The TB notification is influenced by a multitude of interconnected factors, encompassing social determinants of health, healthcare accessibility, community TB prevalence, and demographic characteristics. It is important to note that while particular subgroups may exhibit higher TB notification rates, this is not a universally applicable trend. To gain a precise understanding of TB notification rates among young women, a comprehensive analysis of local epidemiological data and careful consideration of contextual factors that may contribute to such trends are necessary.

The trend in TB notification rate differed significantly according to gender. While the notification rate increased in the last three years among women, it decreased significantly among men. The decline in the notification in 2020 reflects the impact of COVID-19 on the diagnosis and management of TB. One possible reason for decreased notification rate in men over time is that Jordanian men are less likely than women to seek or access care. Factors such as loss of income and financial barriers, as well as stigma, affect men’s healthcare decisions in Jordan. Moreover, the decline in TB notification among men may be due to a low index of suspicion by health practitioners, especially for subclinical TB cases. Interventions to improve case detection among men must recognize and address these barriers.

Nearly half of all the TB cases were among Jordanians and the majority of non-Jordanian cases were among Syrian refugees. Despite the challenges of controlling TB in refugee settings, Jordan’s NTB control program contributed effectively to improving Syrian refugee health, preventing new TB cases, and minimizing the emergence of Multiple Drug Resistance (MDR-TB) [3]. There has been an almost 40% increase in detected TB case rates among Syrian refugees in Jordan since the national TB strategy was implemented in July 2013. Tuberculosis is often an unrecognized disease; thus, screening is essential for early detection and treatment. Of those Syrians screened under the public health strategy, nearly 80% were sputum-smear negative potentially attesting to less risk of transmission of TB among the refugees as well as the host population.

The age-standardized prevalence rate of Latent TB was estimated at 2.71% in Jordan in 2019 [26]. Recent data on the burden of latent TB among the refugee population in Jordan is lacking. Since 2011, the NTP implemented a specific TB reduction strategy including contact tracing in response to the influx of displaced Syrians. Contacts of all refugees diagnosed with pulmonary TB were registered by the International Organization for Migration and screened for active and latent TB in 6 NTP centers. In a retrospective study among contacts of all pulmonary TB cases diagnosed between March 2011 and May 2014, authors found a high prevalence of active TB and latent TB cases among contacts of pulmonary TB cases in the Syrian refugee population [27]. Latent TB was diagnosed in 24.1% of contacts tested while active TB was diagnosed in 2.1% of contacts. In response, the Public Health Strategy for TB among Syrian Refugees in Jordan was developed by the IOM, NTP, and United Nations High Commissioner for Refugees (UNHCR) with technical assistance from the Centers for Disease Control and Prevention (CDC) and WHO-Jordan approval. The public health strategy’s objectives included increasing TB screening among Syrian refugees, increasing TB diagnosis, maximizing treatment success, increasing TB awareness and knowledge of treatment services, and supporting the development and implementation of guidelines for the management of latent TB infections.

## 5. Conclusions

While the notification rate increased in the last three years among women, it decreased significantly among men. There is a need to ensure that the national TB plans set clear targets for reducing the burden of TB. Integrating TB case finding into COVID-19 screening, testing, tracing, and monitoring systems is recommended. Our findings have multiple programmatic implications. Health system and related indicators for diagnosis, treatment, and outcomes of TB should be presented by sex, age, province, nationality, and other stratifiers. Presenting disaggregated data encourages population health researchers and public health planners to consider the intersections of determinants that create or ameliorate inequities for good health and inequalities in health outcomes. Continued efforts are needed to improve the quality of surveillance data, and careful assessment of the accuracy, completeness, and comprehensiveness is required when interpreting the result of the analysis. The MOH should implement an effective strategy so that all health facilities and laboratories that perform tests notify positive cases. Enhancement programs such as health education and patient-awareness augmentation can improve case detection and reduce the time to diagnosis and treatment initiation.

## Figures and Tables

**Table 1:** The demographic and clinical characteristics of patients diagnosed with tuberculosis in Jordan during the period 2016–2020.

Variable	Gender				Total N = 1711	*p*-Value
	Women (*n* = 989)		Men (*n* = 722)			
	*n*	%	*n*	%		
**Age (year)**						<0.001
0–9	40	4.0	52	7.2	92	
10–19	61	6.2	53	7.3	114	
20–29	325	32.9	137	19.0	462	
30–39	302	30.5	139	19.3	441	
40–49	125	12.6	118	16.3	243	
50–59	64	6.5	104	14.4	168	
60–69	37	3.7	62	8.6	99	
≥70	35	3.5	57	7.9	92	
**Marital Status**						0.038
Divorced	6	0.6	1	0.1	7	
Married	678	68.6	464	64.3	1142	
Single	296	29.9	254	35.2	550	
Widow	9	0.9	3	0.4	12	
**Nationality**						<0.001
Non-Jordanian	556	56.2	326	45.2	882	
Jordanian	433	43.8	396	54.8	829	
**Site of TB**						<0.001
Extra-pulmonary	390	39.4	192	26.6	582	
TB Pulmonary	599	60.6	530	73.4	1129	

**Table 2:** The characteristics of patients with TB according to nationality.

Variable	Nationality					
	Jordanians		Syrians		Other Nationalities	
	*n*	%	*n*	%	*n*	%
Gender						
Women	433	52.2	106	40.8	450	72.3
Men	396	47.8	154	59.2	172	27.7
Age (year)						
0–9	54	6.5	25	9.6	13	2.1
10–19	70	8.4	24	9.2	20	3.2
20–29	160	19.3	25	9.6	277	44.5
30–39	170	20.5	61	23.5	210	33.8
40–49	122	14.7	44	16.9	77	12.4
50–59	107	12.9	44	16.9	17	2.7
60–69	76	9.2	19	7.3	4	0.6
≥70	70	8.4	18	6.9	4	0.6
Marital Status						
Divorced	3	0.4	1	0.4	3	0.5
Married	547	66.0	171	65.8	424	68.2
Single	270	32.6	86	33.1	194	31.2
Widow	9	1.1	2	0.8	1	0.2
Year at diagnosis						
2016	183	22.1	56	21.5	71	11.4
2017	206	24.8	74	28.5	47	7.6
2018	153	18.5	62	23.8	167	26.8
2019	179	21.6	35	13.5	235	37.8
2020	108	13.0	33	12.7	102	16.4
**Site of TB**						
Extra-pulmonary	429	51.7	53	20.4	100	16.1
Pulmonary TB	400	48.3	207	79.6	522	83.9

**Table 3:** Gender- and age-specific average annual notification rate of tuberculosis during the period 2016–2020.

	Notification Rate per 100,000 Population		
Age	Women	Men	Total
0–9	0.66	0.82	0.76
10–19	1.2	0.96	1.08
20–29	7.54	2.58	4.8
30–39	8.76	3.44	5.88
40–49	4.9	3.88	4.34
50–59	4.22	6.12	5.22
60–69	4.6	7.46	6.04
70+	6.12	9.56	7.88
Total	4.08	2.64	3.32

## Data Availability

Data are available upon request from the corresponding author.
